# “They treat us equally, they guide us”: peer navigation for HIV care linkage in men who have sex with men and transgender women in Lima, Peru

**DOI:** 10.17843/rpmesp.2024.412.13198

**Published:** 2024-06-13

**Authors:** Andres Maiorana, Elizabeth Lugo, Wendy Hamasaki, Gino Calvo, Kelika Konda, Alfonso Silva-Santisteban, Carlos Cáceres, Susan Kegeles

**Affiliations:** 1 Center for HIV Prevention Studies, University of California, San Francisco, United States. University of California Center for HIV Prevention Studies Universidad de California San Francisco USA; 2 Interdisciplinary Research Center on Sexuality, AIDS and Society, Universidad Peruana Cayetano Heredia. Lima, Peru. Universidad Peruana Cayetano Heredia Interdisciplinary Research Center on Sexuality, AIDS and Society Universidad Peruana Cayetano Heredia Lima Peru

**Keywords:** HIV, Health Services, Peru

## Abstract

**Objective.:**

To analyze the elements of a navigation program in Lima that facilitated the linkage of men who have sex with men (MSM) and transgender women (TW) with HIV care.

**Material and methods.:**

We conducted interviews with 20 users receiving navigation services and 4 peer navigators living with HIV.

**Results.:**

The work of the navigators contributed to filling a gap in HIV services, providing personalized accompaniment to navigate the health care system and facilitating the process of engaging with care.

**Conclusions.:**

Patient navigation based on the development of users’ strengths can be a useful and feasible strategy to improve linkage to medical care for MSM and TW in Peru, incorporating peer navigators to health teams, horizontality in treatment and public health strategies with greater community participation.

## INTRODUCTION

In Peru, as in the rest of Latin America, the HIV epidemic affects mainly men who have sex with men (MSM) and transgender women (TW), with estimated prevalence rates between 10-15% for MSM and 30% for TW ^1^. However, although these populations are disproportionately affected by HIV, their level of linkage with HIV care remains limited ^2^. UNAIDS recommendations indicate that linkage with care and early initiation of antiretroviral therapy (ART) are necessary to achieve viral load undetectability ^3^. An undetectable viral load makes it possible to consider HIV as a chronic disease, to ensure a healthier life for people living with HIV (PLHIV), and to prevent transmission of HIV, referred to as the continuum of prevention and care (CPC) ^4^. However, a nationwide study showed that less than 50% of the total estimated MSM (45%) and TW (43%) living with HIV had evidence of viral suppression ^5^. Causes of lack of viral suppression included: late diagnosis, lack of linkage or inadequate retention in care, and low adherence to treatment ^2^.

Individual-level barriers associated with poor linkage to care for MSM and TW in Peru include: low access to HIV testing, poverty, HIV-related stigma, fear of mistreatment in health facilities because of their sexual identity and gender, and inexperience in a fragmented and unfriendly public health system ^6^. Barriers in the public health system include: long waiting times, indifference among health providers to the sexual and gender diversity of PLHIV, shortages of social workers and psychologists, cumbersome referral processes to specialized services, and limited subsidies for testing. The Antiretroviral Treatment (ART) service and monitoring tests (CD4 and viral load) are free of charge, but the required pre-testing is sometimes difficult to afford ^7^.

In this context, we implemented Project Pride Plus (PO+) ^8^, a multilevel intervention to improve CPC ^4^ in MSM and TW in southern Lima. The intervention included a community mobilization component and another one in health facilities with staff training on sexual diversity, HIV prevention ^9^ and a navigation program which is presented here.

Patient navigation, as a person-centered model of care, is an approach that has proven useful and is increasingly used in different settings, particularly in the US, and in different populations, including MSM and TW, to link PLHIV with healthcare and achieve viral suppression. Navigators provide help and support in navigating complex health systems, acting as a link to medical and other community or support services ^10^^,^^11^^,^^12^. On the other hand, peer navigation incorporates elements of navigator models and the peer role to promote the health and well-being of PLHIV. The peer role is established by having HIV and/or sharing other characteristics with the key population. Peers may serve as paid staff or volunteers in health facilities or other organizations ^13^.

Several navigation programs have been implemented in some Latin American countries to improve linkage to care. In Peru, the results of a nine-month pilot intervention with several individual- and group-level components that included navigation implemented by community health workers conducted from 2019 showed high acceptability among adolescents (aged 18-21 years including MSM and TW) with HIV and health personnel ^14^; as well as its feasibility and evidence of improved adherence, social support, and self-efficacy in these adolescent users ^15^. MSM and TW participants in a study in Guatemala linked with care in an average of three days and expressed that the emotional support of a navigator helped them cope with the fear of their diagnosis ^16^. Social and emotional support from peer navigators was also significant for MSM and TW participants in another intervention in Mexico ^17^. Interventions specifically for TW in Brazil ^18^ and the Dominican Republic ^19^ implemented by peer navigators demonstrated high acceptability and preliminary efficacy in ART adherence. However, limitations in the overall cohesion and trust existing among TW in the Dominican Republic conditioned their approach and involvement with peer navigators and other community mobilization components.

We implemented the PO+ navigation program in two health facilities in southern Lima using the ARTAS (Anti-Retroviral Treatment and Access to Services) methodology, recommended by the Centers for Disease Control in the USA and based on Social Cognitive Theory and the concept of self-efficacy, which has shown promising results ^20^^,^^21^. ARTAS, using a person-centered case approach, was designed to help participants newly diagnosed with HIV use personal strengths to overcome barriers to linkage to care, with the accompaniment of a peer navigator in five sessions over a 90-day period. We contextualized ARTAS for Peru, including people who knew their HIV-positive status without prior treatment and who had dropped out of treatment, with support from navigators for one year on an as-needed basis.

We hired four part-time peer counselors or promoters. Three of them worked in the two health facilities, counseling and providing HIV information. Training them in ARTAS allowed them to expand their role in those facilities to provide navigation services. The fourth person, with no nexus to a health facility, referred MSM and TW from the community for HIV testing or to link or re-link them to care.

For 30 months (July 2017-December 2019), navigators worked with 188 users [150 MSM (80%) and 38 TW (20%)], 160 diagnosed in the last three months, and 28 previously unlinked from medical care, identified through medical records, physician referral, or because navigators already knew them at health facilities or in the community. To initiate the participation process, navigators asked users if they were interested in being part of a health care program and to learn about the functioning of health services. Health care providers also sent users to talk to the navigators. The users were people with low levels of: HIV information, income, social support, and disclosure of their HIV status to others. Of those 188, 170 were linked or re-engaged in care by working with the navigators, who conducted an average of three ARTAS sessions with each user. Navigators met with the study oversight team weekly for monitoring.

A quantitative analysis of the navigator program found that users who received navigator services were statistically more likely to initiate ART within three months, as well as achieve and maintain an undetectable viral load compared with those without navigator services ^22^. As part of the evaluation of the navigation program, this qualitative article discusses elements related to the implementation of the navigation program and how the services, the role of navigators, and their linkage to users helped facilitate linkage of MSM and TW to care. Qualitative research methods are appropriate for understanding the factors that facilitate or inhibit the successful implementation of new interventions and their outcomes ^23^.

**KEY MESSAGES**

Motivation for the study. In Peru, men who have sex with men (MSM) and transgender women (TW) present low levels of linkage to HIV medical care, which is crucial to consider it a chronic disease, guarantee a healthy life and prevent transmission.

Main findings. We implemented a program with specialized personnel called peer navigators, which helped MSM and TW to identify personal strengths and become autonomous within a fragmented and unfriendly health system.

Implications. Incorporating peer navigators is a useful and feasible strategy that contributed to filling a gap in HIV care services, providing accompaniment, education and horizontal treatment to improve linkage to medical care for MSM and TW.

## MATERIALS AND METHODS

### Study design and population

The approach included in-depth interviews using purposive sampling with:

(a) users who worked with navigators, and (b) navigators. We asked navigators to identify MSM and TW of different ages with whom they had worked, with inclusion criteria similar to those of the navigator program: recent diagnosis within the previous three months, old diagnosis with recent linkage, or previous dropout from care. What is reported in this article complies with the Consolidated Criteria for Reporting Qualitative Research (COREQ) guidelines ^24^ (supplementary material).

### Procedures

Inclusion criteria were first applied by the navigators to identify users and then corroborated by the interviewers before beginning the interviews. Once identified, users were invited to participate, explaining the purpose of the interview. None of the contacted users refused to participate in the interviews. The number of interviewed users corresponded to the initial number planned for the purposive sample. The interviews were conducted individually within a private space in the health facilities where the users received care and worked with the navigators. When introducing themselves to the users, whom they did not know previously, the interviewers described the study and their role as a way of creating an atmosphere of trust before beginning the interviews. The navigator interviews were conducted by the coordinator of the community component of the PO+ intervention, an expert in qualitative research. The community component operated separately from the health systems component of PO+. Interviews with users were conducted between February 2019 and January 2020, after they had completed the ARTAS sessions, and with navigators in January-February 2019.

### Data collection techniques and instruments

Two semi-structured guides of questions that integrated dimensions corresponding to the evaluation objectives were used. The interview guide for users included three main dimensions on their experiences working with navigators, barriers to receiving care and treatment, and perceptions related to living with HIV; and for navigators, two main dimensions on their experiences providing navigator services and the barriers and facilitators helping users link to care (supplementary material: Annex 2a and Annex 2b). Prior to its implementation, a small pilot with four PO+ team members allowed refining some questions in the interview guide. The field notes written by the interviewers with relevant points from each interview were useful during the analysis. The interviews lasted approximately 45 minutes, were audio-recorded and transcribed by staff who did not participate in data collection.

### Analysis

The analysis process was iterative and based on thematic analysis ^25^ conducted by the first three authors (AM, ELM, WHR), representing the disciplines of anthropology, psychology, and sociology and with prior experience and training in project evaluation and qualitative research with MSM and TW. Thematic analysis provides a flexible, rich, and detailed approach for examining the perspectives of research participants, highlighting similarities and/or differences and generating themes and subthemes. We avoided the potential limitations of thematic analysis through rigorous analysis, consistency and cohesion in team interpretation of information, and consideration of thematic interrelationship.

A preliminary deductive codebook was developed based on the question guides and inductive codes from the interviews after reading and discussing the transcripts of six interviews; the team met periodically to compare codes and resolve discrepancies. The team then developed two final codebooks (users and navigators) that were applied to all transcripts that were coded using Dedoose ^26^^)^ a web application for organizing data (supplementary material: appendix 3a and appendix 3b). Findings from each interview were summarized after analyzing these codes, examining key themes emerging from the information related to elements of the navigation program implementation. Triangulation of information sources was applied by comparing and integrating information provided by users and navigators. Regardless of the personal characteristics and circumstances associated with each user’s linkage to medical care, the similarity and convergence of their opinions about navigation services and their relationship with navigators, as well as the navigators’ perceptions indicated consistency and saturation in the information, sufficient for the purpose of the research. In addition, the convergence in the perceptions and opinions of users and navigators regarding the navigation program added validity to the results. The analysis included the following steps to ensure its validity, credibility, reliability and confirmability: familiarization with the information; creation of a codebook through team discussion; identification, review and definition of themes as a team, returning to transcript information as necessary; connection and relationship between themes; logbook notes written during periodic team meetings facilitated continuity in methodological decisions and reflexivity during the analysis and writing of results focused on convergent themes ^27^. The level of consensus among different researchers of the same reality raises credibility, as well as the assurance that the level of congruence of the phenomena under study is strong and solid ^28^.

### Ethical Aspects

The Ethics Committees of the University of California at San Francisco (14-15203) and the Universidad Peruana Cayetano Heredia (64195) approved the study. Participants received information about the purpose of the interviews prior to their verbal consent. At the end of the interview, users were given a compensation of 10 PEN (3 USD) for their participation. Navigators were not compensated for participating, as the interview was considered part of their job. We ensured privacy and confidentiality in data handling, and used fictitious names in quotations.

## RESULTS

We analyzed the interviews of 24 participants: 20 users [16 gay men (GM) (80%) and 4 trans women (TW) (20%), percentages that reflect the sample with which the navigators worked], between 20 and 49 years old; seven with recent diagnosis, eight with previous diagnosis and recent treatment, and five with previous diagnosis who abandoned treatment (supplementary material: Annex 4); and four navigators between 50 and 60 years old (two GM and two heterosexual women). The interviewed users were linked to care and were receiving ART. We present the results in three categories: 1) services offered by the navigators; 2) navigator-user relationship as a support tool; and 3) articulation of the navigator’s role with health personnel. We use the term gay referring to the male users interviewed because many of them identified themselves as such.

### Services offered by navigators

The ARTAS training helped navigators develop new skills, providing them with tools to help users identify self-identify strengths and overcome barriers to linking to HIV care. One of them explained: 

*Before we had limitations, we worked on adherence, not missing check-ups, condom use; we evaluated a few things, we didn’t go beyond that.... Now we analyze the problems the person has, what hinders their treatment, what prevents them from returning, we work together with them...* (Navigator 2).

The work of the navigators was characterized by person-centered care, avoiding judgment, respecting the dignity, rights and particularity of each user. Navigator services included:

*Health Education*

HIV education helped dispel ignorance and myths about the diagnosis and promote the normalization of HIV status as a manageable chronic disease and not a death sentence. Users expressed that the navigators provided information about the efficacy of ART emphasizing that current antiretrovirals have fewer side effects and the importance of keeping medical appointments, illustrating with their experience how to accept an HIV-positive diagnosis, incorporating healthy habits and adhering to treatment. 

One user related what she had learned during the navigation:

*The world is not ending, she told me, you are not the only one, as long as the medications are there keep going, she tells me, you are going to have a long life* (Jessica, 45 years old, TW, diagnosis with abandonment).

*Navigation and familiarization with the functioning of health systems*

Many of the interviewed users identified the lack of familiarity with the functioning of health facilities as an obstacle, stating that it was very complex, with complicated processes and long waits. Thus, one user mentioned why she had abandoned her care in the past:

*... when I was diagnosed, I wanted to start treatment, I stayed for sputum test, because as I had a business* [*and did not have time*]*, I was summoned every day to leave the samples, so every day I did not have the time and I left* (Anita, 28 years old, MT, previous diagnosis).

The work of the navigator, as specialized personnel dedicated to these services, contributed to familiarize users with the functioning of the health system, by accompanying them in and out of the services, reminding them of appointments and answering their questions. Thus, the aforementioned user explained that:

*The guys* [*navigators*] *give you encouragement, confidence that you can go, accompany you, help you make your appointment. There is a lot of difference, because at the hospital nobody calls you, if you go, then good, if don’t, you die* (Anita, 28 years old, MT, previous diagnosis).

Some users also commented that working with the navigators facilitated their personal growth and acceptance of the diagnosis, developing autonomy, responsibility and independence to move more easily within the system and continue being involved by themselves in care services, which caused that the contact with the navigators to be less and less frequent each time, not for support but for reasons of friendship. When asked how working with the navigator helped them, one of the users expressed: 

*... from the help they gave me I learned to be independent, that I was not always going to need* [*navigators*]*, there comes a stage, when one already has to get on track and make one’s own way, more than counselors they are my friends* (Pedro, 38 years old, GM, recent diagnosis). 

A navigator, in a similar way and reflecting the convergence with what was expressed by the users, exemplified how she perceived the process of adaptation and empowerment of the users within the health system. 

*When they start coming regularly, and they only call to “I’m out of the appointment”; “I’ve already picked up my medications”; “I’m here to say hello”; I consider that they don’t need me, they are on their way* (Navigator 1).

*Advocacy to facilitate care*

Their knowledge of health services and health personnel enabled navigators to help users learn more about how health services work and reduce obstacles such as lack of counseling to obtain health insurance, waiting hours, and the cumbersome process for referrals. As part of his advocacy work, a navigator explained how he interceded on behalf of users:

*If somebody misses an appointment, the one who helps us to reschedule is the social worker. I tell her: “this guy didn’t come because he’s working, can he come on Saturday?” Even if it’s on an additional list, but they put us in, they give us all the facilities* (Navigator 1).

As part of, but also beyond their assigned functions, reflecting commitment to the need, the navigators facilitated obtaining economic support so that users could pay for complementary tests to enter the ART program, interceding for a reduction in the cost of the tests, seeking support from external institutions or helping with admission to the Comprehensive Health Insurance (SIS). In some cases, they raised money for users to start a small business to generate capital and cover expenses. One navigator explained:

*We have patients who don’t even have enough money for previous testing. I have had to find them an organization that can pay for them. We have tried to get things together* [*navigator and nurses*] *to make a “pollada” and make income with their profit, and buy thermoses* [*for selling coffee on the street*]*. Now they are doing well, they are self-supporting. This goes beyond ARTAS. ARTAS does not give us money to give them...* (Navigator 2). 

Similarly, one of the users stated:

*Yes, he helped me in everything* [*navigator*]*. Since the tests, he contacted me with the program of ¿what's its name?... UN. Because I had no job, nothing, so it was expensive for me to pay for the series of tests* (Luis, 32 years old, GM, previous diagnosis).

### Navigator-user relationship as a support tool

The relationship established between navigators and users created emotional bonds based on trust and support that facilitated the linkage or re-linkage of GM and TW to care and treatment. This relationship was characterized by:

*Horizontal relationships*

The navigators, through an empathetic attitude and active listening techniques, fostered horizontal relationships that facilitated collaboration with users. This led to the exchange of experiences in accompanying users, promoting an active role in linking or re-linking them to treatment, and making them aware of the importance of adherence to treatment. Miguel, for example, remarked on the personalized and affectionate treatment he had received from the navigator:

*... the trust and love for each and every patient... I go to another office and not even the nurse greets you, or anything. It’s not the same as* [*name of navigator*]*, “welcome, come here, we’ll look for you here, my friend”, and it’s that empathy.* (Miguel, 28 years old, GM, previous diagnosis).

*Emotional and social support*

As part of the relationship established with the users, the emotional and social support provided by the navigators during the ARTAS sessions and during the accompaniment facilitated bonding with the user. This bonding allowed users to express their feelings, helped to clarify their emotional experiences and to understand that their reactions were valid. This process also helped to develop self-efficacy in the users to cope with and accept their diagnosis.

The navigators addressed a variety of negative attitudes toward HIV in users stemming from internalized stigma, which influenced how they perceived themselves and how they believed they were perceived by their environment. Internalized stigma caused users to be afraid or ashamed to disclose their diagnosis, and problems in adherence to treatment in situations of living with people who were unaware of their HIV status. The need to hide their diagnosis made the navigators the only affective and supportive network for the users. Thus, the latter showed great satisfaction with the support of the navigators in moving forward, and said they trusted them to tell them about their concerns or experiences. Most of them established strong emotional ties with the navigators, referring to them as friends, family or “someone sent from heaven”. 

*I consider her as a friend, like a mom. She* [*navigator*] *supported me in everything. I tell her “thanks to you I'm fine”* (Ricardo, 22 years old, GM, recent diagnosis).

*Importance of the peer role of navigators*

Users expressed greater comfort with the navigators’ peer status for having HIV, or in the case of male navigators, for sharing their sexual identity. This allowed them to perceive the navigator as a role model and to learn, through their experiences, strategies to replace “the world is coming down” with “I can be okay too.” The experiences of the navigators, living with HIV for a long time, and perhaps also their age, allowed the users to reduce their feelings of loneliness, guilt and fatalism in the face of HIV. Peter, quoted in the title, referring to sharing his sexual identity with the navigator, but also implicitly to living with HIV, expressed: 

*Because he is like us, he understands us. He's our gay dad, he treats us equally, he guides us. If I know someone who has the same diagnosis, I would tell him to come, that I have* [*navigator*] *there...he is going to help you, he is going to be your friend* (Pedro, 28 years old, GM, previous diagnosis).

*Obstacles to the work of navigators*

Despite the effectiveness of their work and the good relationships established with the users, in some cases the navigators expressed frustration with certain barriers evidenced in the users, such as drug use (cocaine) or the values of some of them or their families who considered HIV as “God’s punishment”. These behaviors and beliefs led to attitudes, feelings of guilt and other negative emotions that prevented normalization of the diagnosis, acceptance of their sexuality or linkage to care. One navigator described family rejection as an obstacle:

*Their family didn't know he was gay and got infected, he wasn’t going to undergo treatment so they wouldn't find out. I had to talk to the mom from my experience. Until now she says “I will not forgive him”, but whenever there is a* [*medical*] *visit she always accompanies him. It was difficult because sometimes religions weigh a lot* (Navigator 2).

### Articulation of the role of the navigator with the health personnel

The navigators, through their work, developed or strengthened their articulation with the rest of the health personnel. This allowed them to shorten procedures for users, facilitate the booking of medical appointments, improve follow-up and help strengthen the doctor-patient relationship. It is worth noting that, although at the beginning of the intervention PO+ organized sensitization training for health personnel on the treatment and needs of GM and TW, neither PO+ nor the health centers implemented changes to facilitate patient flow, procedures or appointment booking. Only the navigators fulfilled that role.

Navigators stated that health personnel showed satisfaction and openness towards their work, trusting them in their role and asking them to orient users about health services: 

*Doctors tell me “We need you to talk to a new person, help him understand the system”. I talk to him; I see his difficulty. We start with ARTAS, let him see its potential* (Navigator 2).

The navigators played the role of a link between health providers and users. For example, they communicated to health providers concerns or problems that users often avoided addressing because of shyness or fear of being judged. One navigator spoke of how she encouraged them to communicate better with their provider:

*I motivate them: “Tell her the truth, the doctor won’t be upset. Doctors understand that these are intimate things.” I sensitize them, because they think “how embarrassing, she’s going to look at me* [*genito-anal parts*]*”* (Navigator 1).

## DISCUSSION

Our results suggest that navigation implemented by peer navigators may be a useful strategy to improve bonding in MSM and TW CPC in Peru. Being that peer navigation represents a complex social-level intervention whose elements or processes have not been adequately examined ^13^, the present study’s contribution to the literature is to analyze the elements related to the implementation of the PO+ navigation program in South Lima that facilitated or influenced linkage to medical care.

The provided services and HIV education, the navigators’ supportive relationship with users, and the articulation of their role with the rest of the health personnel, main axes and themes of our results, helped GM and TW to normalize HIV status and facilitated their linkage and re-linkage to HIV health services. The navigator services and functions were characterized by emotional support and personalized accompaniment so that users could identify or develop strengths and overcome barriers to guide themselves within the health system. Navigators developed trusting relationships that promoted empowerment, self-efficacy, and played a protective role that helped enhance users’ resilience and became role models for health care and treatment adherence while living with HIV. Likewise, the results reinforce the importance of the role and influence of peers in sharing similar characteristics or experiences conducive to greater empathy and promotion of healthy behaviors ^29^ where, in this case GM and TW, take an active role in their own care living with HIV. The work of the navigators helped to fill a gap in the often collapsed and unfriendly HIV care services, so that users would have a person with dedicated time and effort who understood them to guide them through the process of linkage to care. In addition, the results suggest the feasibility of incorporating peer navigators into health teams with functions besides those of promoters or peer counselors, facilitating the functioning of services and the work of medical staff and contributing to greater horizontality and community participation in HIV services ^30^.

Navigators helped users to navigate the health system, but the systemic barriers already mentioned in the introduction that hinder linkage to care still persist. However, by promoting an active role for health care and developing awareness of the reality of the services, this navigator program can contribute to the empowerment of PLHIV and their linkage to care. An empowerment model implies building a process of user involvement and commitment to their health, having adequate information and personal resources to evaluate such information, in collaboration with health care providers, and with sufficient tolerance of uncertainty regarding medical care ^31^.

This study has some limitations. Consistent with qualitative methods, i.e., our results can not be generalized to all users with HIV, but are similar to what has been found, for example, by other studies of GM navigation programs in Guatemala and with populations of Latino origin in the U.S. that included GM and TW ^32^^,^^33^. The interviewed users were linked to medical care, so we have no evidence of the perceptions of MSM and TW who did not link to or dropped out of care. The sample of users was purposive, and participants were identified by the navigators, but the inclusion criteria were corroborated by the interviewers with the users before conducting the interviews. Although a difficulty of the thematic analysis could be to identify and prioritize relevant topics and subtopics, we avoided this barrier through rigorous analysis, thematic interrelation, and consistency and cohesion in the team interpretation of the information. The navigator-user link could be affected for different reasons, although this should be avoided based on the training received by the navigators, considering that the navigation services are designed for a certain duration and to be centered on the users and the development of their strengths and self-efficacy, resulting in their eventual independence from the navigators. Finally, we did not include the perceptions from the health care team and medical providers about the navigation program, which could be explored by future studies.

Navigation services may have potential programmatic and cost value. Future research could specifically examine the feasibility, acceptability, and sustainability of navigation services in Peru, as well as the potential cost savings resulting from such a program. Potential and challenging next steps would be to seek resources to integrate navigation services for PLHIV in health centers in Peru. Although few studies of navigation services in the U.S. have included cost analyses, their results indicate that such services were cost-effective or cost-saving. Their inclusion in health services in Peru would also merit an analysis of their potential cost and effectiveness compared to current standard services ^34^^,^^35^. Likewise, this navigation program could be applied in other Latin American countries with an HIV epidemic also concentrated in MSM and TW.
