# Supplementary material for: “They treat us equally, they guide us”: peer navigation for HIV care linkage in men who have sex with men and transgender women in Lima, Peru
Source: Rev Peru Med Exp Salud Publica. 2024 Jun 13;41(2):105–13. doi: 10.17843/rpmesp.2024.412.13198 (PMC11300691; doi:10.17843/rpmesp.2024.412.13198)
Supplement: Translation

# Translation: «Nos trata por igual, nos guía»: navegación de pares para la vinculación al cuidado del VIH en hombres que tienen sexo con hombres y mujeres transgénero en Lima, Perú

**Authors:** Andres Maiorana, Elizabeth Lugo, Wendy Hamasaki, Gino Calvo, Kelika Konda, Alfonso Silva-Santisteban, Carlos Cáceres, Susan Kegeles

**Affiliations:** 1 Centro de Estudios para la Prevención del VIH, Universidad de California, San Francisco, Estados Unidos.; 2 Centro de Investigación Interdisciplinaria en Sexualidad, Sida y Sociedad, Universidad Peruana Cayetano Heredia. Lima, Perú.

**Keywords:** VIH, Servicios de Salud, Perú

## Abstract

**Objetivo.:**

Analizar los elementos de un programa de navegación en Lima que facilitaron la vinculación de hombres que tienen sexo con hombres (HSH) y mujeres transgénero (MT) a la atención del VIH.

**Materiales y métodos.:**

Realizamos entrevistas a 20 usuarios recibiendo servicios de navegación y a 4 navegadores pares viviendo con VIH.

**Resultados.:**

El trabajo de los navegadores contribuyó a cubrir una brecha en los servicios para el VIH, brindando acompañamiento personalizado para navegar el sistema de salud y facilitando el proceso de vinculación a la atención médica.

**Conclusiones.:**

La navegación de pacientes basada en el desarrollo de fortalezas de los usuarios puede ser una estrategia útil y factible para mejorar la vinculación al cuidado médico de HSH y MT en Perú, incorporando navegadores pares a los equipos de salud, horizontalidad en el trato y estrategias de salud pública con mayor participación comunitaria.

## INTRODUCCIÓN

En Perú, similarmente al resto de América Latina, la epidemia del VIH se encuentra concentrada principalmente en hombres que tienen sexo con hombres (HSH) y mujeres trans (MT), con prevalencias estimadas entre 10-15% en HSH y 30% en MT ^1^. No obstante, aunque estas poblaciones están desproporcionadamente afectadas por el VIH, su nivel de vinculación a la atención médica del VIH continúa siendo limitado ^2^. Las recomendaciones de ONUSIDA indican que la vinculación a la atención médica y el inicio temprano del tratamiento antirretroviral (TAR) son necesarios para obtener la indetectabilidad de la carga viral ^3^. Una carga viral indetectable permite considerar al VIH como una enfermedad crónica, garantizar una vida más saludable a las personas viviendo con VIH (PVV) y prevenir la transmisión del VIH a otras personas, lo que se denomina el continuo de prevención y cuidado (CPC) ^4^. Sin embargo, un estudio a nivel nacional indicó que menos del 50% del total de los estimados HSH (45%) y MT (43%) que viven con VIH tenían evidencia de supresión viral ^5^. Las causas de ausencia de supresión viral incluían: diagnóstico tardío, falta de vinculación o retención inadecuada a la atención y baja adherencia al tratamiento ^2^.

Las barreras a nivel individual asociadas con la poca vinculación a la atención en HSH y MT en Perú incluyen: bajo acceso a la prueba VIH, pobreza, estigma en relación al VIH, temor al maltrato en establecimientos de salud por su identidad sexual y género e inexperiencia en un sistema de salud pública fragmentado y poco amigable ^6^. Las barreras a nivel del sistema de salud pública incluyen: prolongado tiempo de espera, indiferencia entre los proveedores de salud ante la diversidad sexual y de género de PVV, escasez de trabajadores sociales y psicólogos, procesos engorrosos para derivaciones a servicios especializados y subsidios limitados para análisis. El servicio de Tratamiento Antiretroviral (TAR) y las pruebas de monitoreo (CD4 y carga viral) son gratuitas pero los exámenes previos requeridos tienen un costo a veces difícil de solventar ^7^.

Bajo este contexto, implementamos Proyecto Orgullo Plus (PO+) ^8^, una intervención multinivel para mejorar el CPC ^(^^4^ en HSH y MT en Lima Sur. La intervención incluyó un componente de movilización comunitaria y otro en establecimientos de salud con capacitaciones al personal sobre diversidad sexual, prevención del VIH ^9^ y un programa de navegación que aquí presentamos.

La navegación de pacientes, como un modelo de atención centrado en la persona, es un enfoque que ha demostrado utilidad y que es cada vez más usado en diferentes lugares, particularmente en EE. UU, y con distintas poblaciones, incluyendo HSH y MT, para vincular a PVV a la atención médica y lograr la supresión viral. Los navegadores proveen ayuda y apoyo para navegar sistemas de salud complejos, actuando como vínculo con servicios médicos y otros servicios comunitarios o de apoyo ^10^^,^^11^^,^^12^. Por otra parte, la navegación de pares incorpora elementos de modelos de navegación y el rol de pares para promover la salud y el bienestar de PVV. El rol de par se establece por tener VIH y/o por compartir otras características con la población clave. Los pares pueden desempeñarse como personal pago o voluntarios en establecimientos de salud u otras organizaciones ^13^.

Varios programas de navegación se han aplicado en algunos países latinoamericanos para mejorar la vinculación a la atención. En Perú, los resultados de una intervención piloto de nueve meses, con varios componentes a nivel individual y grupal que contemplaban navegación implementada por trabajadores de salud comunitarios realizada a partir del 2019, demostraron una alta aceptabilidad entre adolescentes (de 18 a 21 años que incluían HSH y MT) con VIH y el personal de salud ^14^; así como también su factibilidad y evidencia de mejora en la adherencia, apoyo social y autoeficacia en dichos usuarios adolescentes ^15^. Participantes HSH y MT en un estudio en Guatemala fueron vinculados a la atención en una media de tres días y expresaron que el apoyo emocional de un navegador les ayudó a afrontar el temor frente a su diagnóstico ^16^. El apoyo social y emocional de navegadores pares fue significativo también para participantes HSH y MT en otra intervención en México ^17^. Intervenciones específicamente para MT en Brasil ^18^ y República Dominicana ^19^ implementadas por navegadores pares demostraron una alta aceptabilidad y una eficacia preliminar en la adherencia a TAR. Sin embargo, limitaciones en la cohesión y confianza existentes en general entre MT en la República Dominicana condicionaron su acercamiento e involucramiento con las navegadoras pares y otros componentes de movilización comunitaria.

Implementamos el programa de navegación de PO+ en dos establecimientos de salud de Lima Sur usando la metodología ARTAS (Anti-Retroviral Treatment and Access to Services), recomendada por los Centros de Control de Enfermedades en EE. UU y basada en la Teoría Social Cognitiva y el concepto de autoeficacia, la cual ha demostrado resultados prometedores ^20^^,^^21^. ARTAS, utilizando un enfoque de casos centrado en la persona, fue diseñado para ayudar a usuarios recién diagnosticados con VIH a utilizar fortalezas personales para superar barreras a la vinculación a la atención, con el acompañamiento de un navegador par en cinco sesiones administradas en un período de 90 días. Contextualizamos ARTAS para Perú, incluyendo a personas que conocían su estatus seropositivo sin tratamiento previo y que habían abandonado el tratamiento, con apoyo de los navegadores durante un año de acuerdo a la necesidad.

Contratamos a medio tiempo a cuatro consejeros de pares o promotores. Tres de ellos ya trabajaban en los dos establecimientos de salud, orientando y brindando información sobre el VIH. Capacitarles en ARTAS permitió expandir su rol en esos establecimientos para proveer servicios de navegación. La cuarta persona, sin nexo con un establecimiento de salud, derivaba a HSH y MT de la comunidad para hacerse la prueba del VIH o para vincularles o revincularles a la atención médica.

Por 30 meses (julio 2017-diciembre 2019), los navegadores trabajaron con 188 usuarios [150 HSH (80%) y 38 MT (20%)], 160 diagnosticados en los últimos tres meses, y 28 previamente desvinculados de la atención médica, identificados a través de registros médicos, derivación de los médicos o porque los navegadores ya los conocían en los servicios de salud o en la comunidad. Para iniciar el proceso de participación, los navegadores preguntaban a los usuarios si les interesaba ser parte de un programa para el cuidado de la salud y para conocer el funcionamiento de los servicios de salud. Los proveedores de salud también enviaban a los usuarios a hablar con los navegadores. Los usuarios eran personas con niveles bajos de: información sobre el VIH, ingresos económicos, apoyo social y divulgación de su estado del VIH a otras personas. De esos 188, 170 se vincularon o revincularon al cuidado médico al trabajar con los navegadores, quienes realizaron un promedio de tres sesiones de ARTAS con cada usuario. Los navegadores se reunían con el equipo de supervisión del estudio semanalmente para monitoreo.

Un análisis cuantitativo del programa de navegación encontró que estadísticamente era más probable que los usuarios que recibieron servicios de navegación iniciaran TAR en tres meses, alcanzaran y mantuvieran una carga viral indetectable comparados con aquellos sin servicios de navegación ^22^. Como parte de la evaluación del programa de navegación, el presente artículo cualitativo analiza los elementos relacionados con la implementación de dicho programa y cómo los servicios, la función de los navegadores y su vínculo con los usuarios ayudaron a facilitar la vinculación de HSH y MT a la atención. Los métodos de investigación cualitativos son apropiados para comprender los factores que facilitan o inhiben la implementación exitosa de nuevas intervenciones y sus resultados ^23^.

**MENSAJES CLAVE**

Motivación para realizar el estudio. En Perú, los hombres que tienen sexo con hombres (HSH) y mujeres transgénero (MT) presentan bajos niveles de vinculación a la atención médica del VIH, crucial para considerarla una enfermedad crónica, garantizar una vida saludable y prevenir la transmisión.

Principales hallazgos. Implementamos un programa con personal especializado llamados navegadores pares, que ayudó a HSH y MT a identificar fortalezas personales y ser autónomos dentro de un sistema de salud fragmentado y poco amigable.

Implicancias. Incorporar navegadores pares es una estrategia útil y factible que contribuyó a cubrir una brecha en los servicios de atención para el VIH, proporcionando acompañamiento, educación y trato horizontal para mejorar la vinculación al cuidado médico de HSH y MT.

## MATERIALES Y MÉTODOS

### Diseño y población del estudio

El enfoque empleado incluyó entrevistas en profundidad utilizando un muestreo intencional con: (a) usuarios que trabajaban con los navegadores, y (b) navegadores. Solicitamos a los navegadores que identificaran HSH y MT de distintas edades con los que habían trabajado, con criterios de inclusión similares a los del programa de navegación: diagnóstico reciente en los tres meses previos, diagnóstico antiguo con vinculación reciente o previo abandono de la atención. Lo reportado en este artículo se ajusta a las directrices del Consolidated Criteria for Reporting Qualitative Research (COREQ) ^24^ (material suplementario).

### Procedimientos

Los criterios de inclusión fueron primero aplicados por los navegadores para identificar a usuarios y después corroborados por las entrevistadoras antes de comenzar las entrevistas. Una vez identificados, se invitó a los usuarios a participar, explicándoles el propósito de la entrevista. Ninguno de los usuarios contactados se negó a participar en las entrevistas. El número de usuarios entrevistados correspondió con el número inicial propuesto para la muestra intencional. Las entrevistas fueron conducidas en forma individual dentro de un espacio privado en los establecimientos de salud donde los usuarios recibían atención y trabajaban con los navegadores. Al presentarse ante los usuarios, a quienes no conocían previamente, las entrevistadoras describían el estudio y su rol como forma de crear un ambiente de confianza antes de iniciar las entrevistas. Las entrevistas a los navegadores fueron realizadas por la coordinadora del componente comunitario de la intervención de PO+, experta en investigación cualitativa. El componente comunitario funcionaba de forma separada al componente de sistemas de salud de PO+. Las entrevistas con usuarios se realizaron entre febrero 2019 y enero 2020, después de que habían completado las sesiones de ARTAS, y con navegadores en enero-febrero 2019. 

### Técnicas e instrumentos de recolección de información

Se emplearon dos guías semiestructuradas de preguntas que integraban dimensiones correspondientes con los objetivos de la evaluación. La guía de entrevistas para usuarios incluía tres dimensiones principales sobre sus experiencias trabajando con los navegadores, barreras para recibir atención y tratamiento y percepciones relacionadas a vivir con VIH; y para navegadores, dos dimensiones principales sobre sus experiencias proporcionando servicios de navegación y las barreras y facilitadores ayudando a usuarios a vincularse al cuidado (material suplementario: anexo 2a y anexo 2b). Previamente a su aplicación, un pequeño piloto con cuatro miembros del equipo de PO+ permitió afinar algunas preguntas en la guía de entrevistas. Las notas de campo redactadas por las entrevistadoras con puntos relevantes de cada entrevista fueron útiles en el proceso de análisis. Las entrevistas duraron aproximadamente 45 minutos, fueron grabadas en audio y transcripción por personal que no participó en la recolección de datos.

### Análisis

El proceso de análisis fue iterativo y basado en análisis temático ^(^^25^ realizado por los primeros tres autores (AM, ELM, WHR), representando las disciplinas de antropología, psicología y sociología y con experiencia y capacitación previa en evaluación de proyectos e investigación cualitativa con HSH y MT. El análisis temático provee un enfoque flexible, rico y detallado para examinar las perspectivas de participantes en la investigación, realzando similitudes y/o diferencias y generando temas y subtemas. Evitamos las posibles limitaciones del análisis temático a través de un análisis riguroso, consistencia y cohesión en la interpretación en equipo de la información y teniendo en cuenta la interrelación temática.

Después de leer y discutir las transcripciones de seis entrevistas, se elaboró un libro preliminar de códigos deductivos basado en las guías de preguntas e inductivos surgidos de las entrevistas y el equipo se reunió periódicamente para comparar códigos y resolver discrepancias. Luego, el equipo desarrolló dos libros de códigos finales (usuarios y navegadores) que se aplicó a todas las transcripciones que fueron codificadas utilizando Dedoose ^26^ una aplicación web para organizar datos (material suplementario: anexo 3a y anexo 3b). Al analizar dichos códigos, se resumieron los hallazgos de cada entrevista, examinando temas claves emergentes de la información relacionada con los elementos de la implementación del programa de navegación. Se aplicó triangulación de las fuentes de información al comparar e integrar la información provista por usuarios y navegadores. Independientemente de las características personales y circunstancias asociadas a la vinculación a la atención médica de cada usuario, la similitud y convergencia de sus opiniones sobre los servicios de navegación y su relación con los navegadores, así como las percepciones de los navegadores indicaron consistencia y saturación en la información, suficiente para el propósito de la investigación. Además, la convergencia en las percepciones y opiniones de usuarios y navegadores con respecto al programa de navegación agregó validez a los resultados. El análisis incluyó los siguientes pasos para asegurar su validez, credibilidad, confiabilidad y confirmabilidad: familiarización con la información; creación de un libro de códigos a través de discusión del equipo; identificación, revisión y definición de temas en equipo, retornando a la información de las transcripciones de acuerdo a lo necesario; conexión y relación entre temas; notas en una bitácora redactadas durante las reuniones periódicas de equipo facilitaron continuidad en las decisiones metodológicas y la reflexibilidad durante el análisis y redacción de los resultados enfocados en temas convergentes ^27^. El nivel de consenso entre diferentes investigadores de la misma realidad eleva la credibilidad, así como la seguridad de que el nivel de congruencia de los fenómenos en estudio sea fuerte y sólido ^28^.

### Aspectos éticos

Los Comités de Ética de la Universidad de California en San Francisco (14-15203) y la Universidad Peruana Cayetano Heredia (64195) aprobaron el estudio. Los participantes recibieron información sobre el propósito de las entrevistas previo a su consentimiento verbal. Al finalizar la entrevista se les dio a los usuarios una compensación de 10 PEN (3 USD) por su participación. No se compensó a los navegadores por participar, por considerarse la entrevista parte de su trabajo. Aseguramos la privacidad y confidencialidad en el manejo de los datos, y usamos nombres ficticios en las citas.

## RESULTADOS

Analizamos las entrevistas de 24 participantes: 20 usuarios [16 hombres gais (HG) (80%) y 4 mujeres trans (MT) (20%), porcentajes que reflejan la muestra con la que trabajaron los navegadores], entre 20 a 49 años; siete con diagnóstico reciente, ocho con diagnóstico antiguo con tratamiento reciente, y cinco con diagnóstico antiguo con abandono de tratamiento (material suplementario: anexo 4); y cuatro navegadores entre 50 a 60 años (dos HG y dos mujeres heterosexuales). Los usuarios entrevistados estaban vinculados a la atención y recibían TAR. Presentamos los resultados en tres categorías: 1) servicios ofrecidos por los navegadores; 2) relación navegador-usuario como herramienta de soporte y 3) articulación del rol del navegador con el personal de salud. Utilizamos el término gay refiriéndonos a los usuarios hombres entrevistados porque muchos de ellos así se identificaban.

### Servicios ofrecidos por los navegadores

La capacitación en ARTAS ayudó a los navegadores a desarrollar nuevas destrezas, proporcionándoles herramientas para ayudar a los usuarios a identificar fortalezas propias y superar barreras para vincularse a la atención del VIH. Uno de ellos explicó:

*Antes teníamos limitaciones, trabajábamos adherencia, que no falten a controles, uso del condón; evaluábamos algunas cosas, no íbamos más allá... Ahora analizamos los problemas que tiene la persona, qué obstaculiza su tratamiento, qué hace que no pueda regresar, trabajamos conjuntamente con ellos...* (Navegador 2).

El trabajo de los navegadores se caracterizó por una atención centrada en la persona, evitando juzgarla, respetando la dignidad, derechos y particularidad de cada usuario. Los servicios de navegación incluyeron:

*Educación para la Salud*

La educación sobre el VIH ayudó a disipar el desconocimiento y mitos sobre el diagnóstico y promover la normalización de la condición de VIH como una enfermedad crónica manejable y no una sentencia de muerte. Los usuarios expresaron que los navegadores brindaban información sobre la eficacia del TAR enfatizando que los antirretrovirales actuales tienen menos efectos secundarios y la importancia de acudir a las citas médicas, ilustrando con su experiencia cómo aceptar un diagnóstico seropositivo, incorporando hábitos saludables y adherirse al tratamiento. Una usuaria relató qué aprendizaje había internalizado durante la navegación:

*El mundo no se acaba, me decía, tú no eres la única, mientras que estén los medicamentos ahí sigue pa´ adelante me dice, vas a tener una vida larga* (Jessica, 45 años, MT, diagnóstico con abandono) 

*Navegación y familiarización con el funcionamiento de los sistemas de salud*

Muchos de los usuarios entrevistados manifestaron como obstáculo el desconocimiento del funcionamiento de los establecimientos de salud, expresando que era muy complejo, con procesos complicados y largas esperas. Así, una usuaria refirió por qué ella había en el pasado abandonado su atención:

*… cuando me diagnostican, yo quería empezar el tratamiento, me quedé en prueba de esputo, porque como tenía negocio [y no contaba con tiempo], me citaban todos los días para dejar las muestras, entonces yo todos los días como que no me daba el tiempo y lo dejé»* (Anita, 28 años, MT, diagnóstico antiguo).

El trabajo del navegador, como personal especializado y dedicado a estos servicios, contribuyó a que los usuarios pudieran familiarizarse con el funcionamiento del sistema de salud, a través del acompañamiento dentro y fuera de los servicios, recordatorios de citas y respondiendo consultas. Así, la usuaria recién mencionada, detalló que:

*Los chicos [navegadores] te dan ánimos, confianza de que puedas ir, te acompañan, ayudan a sacar tu cita. Hay mucha diferencia, porque en el hospital nadie te llama, si vas bueno, si no, te mueres* (Anita, 28 años, MT, diagnóstico antiguo).

Algunos usuarios también comentaron que el trabajar con los navegadores facilitó su crecimiento personal y aceptación del diagnóstico, desarrollando autonomía, responsabilidad e independencia para desplazarse con mayor facilidad dentro del sistema y continuar por sí solos involucrados en los servicios de atención, generando que el contacto con los navegadores fuera cada vez menos frecuente y no para búsqueda de apoyo sino por motivos de amistad. Ante la pregunta de cómo el trabajo con el navegador les ayudó, uno de los usuarios expresó:

*… desde la ayuda que me dieron aprendí a ser independiente, a que no siempre iba a necesitar de [navegadoras], llega una etapa, en que uno ya tiene que enrumbar y hacer su camino, más que consejeras son mis amigas* (Pedro, 38 años, HG, diagnóstico reciente).

Una navegadora, en forma similar y reflejando la convergencia con lo expresado por los usuarios, ejemplificó cómo percibía el proceso de adaptación y empoderamiento de los usuarios dentro del sistema de salud.

*Cuando empiezan a venir regularmente, y solo llaman para ‘ya salí de mi consulta’; ‘ya recogí mis medicamentos’; ‘estoy aquí para saludarte’; considero que no me necesitan, están encaminados* (Navegadora 1).

*Abogacía para facilitar la atención*

Su conocimiento de los servicios de salud y del personal de salud permitió a los navegadores ayudar a los usuarios a conocer mejor el funcionamiento de los servicios de salud y reducir obstáculos como la falta de asesoría para obtener seguro médico, las horas de espera y el proceso engorroso para derivaciones a interconsultas. Como parte de su trabajo de abogacía, un navegador explicó como intercedía de parte de los usuarios:

*Si un chico pierde la cita, la que nos facilita para reprogramar es la asistenta social. Le digo: ‘este chico no vino porque trabaja ¿puede venir el sábado?’ Aunque sea en una lista adicional, pero nos colocan, nos dan todas las facilidades* (Navegador 1).

Como parte de, pero también más allá de las funciones asignadas, reflejando compromiso frente a la necesidad, los navegadores facilitaron conseguir apoyo económico para que los usuarios pudieran costear los análisis complementarios para ingresar al programa TAR, intercediendo para que les redujeran el costo de los análisis, buscando apoyo de instituciones externas o ayudando con el ingreso al Seguro Integral de Salud (SIS). En algunos casos, recaudaron dinero para que los usuarios iniciaran un pequeño emprendimiento para generar capital y cubrir gastos. Un navegador explicó:

*Tenemos pacientes que no tienen ni para exámenes previos. He tenido que buscarles una organización que pueda costearlos. Hemos tratado de juntar cosas [navegador y enfermeras] para hacer una pollada y con su ganancia hagan su capital, y compren termos [vendiendo café en la calle]. Ahora están bien, se costean solos. Eso va más allá de ARTAS. ARTAS no da plata para darles…* (Navegador 2).

De forma similar, uno de los usuarios manifestó:

*Sí, me ayudó en todo [navegador]. Desde los exámenes, me contactó con el programa de ¿cómo se llama?... ONU. Porque no tenía trabajo, nada, entonces me era costoso pagar la serie de exámenes (Luis, 32 años, HG, diagnóstico antiguo).*

### Relación navegador-usuario como herramienta de soporte

La relación establecida entre navegadores y usuarios creó lazos emocionales basados en la confianza y el apoyo que facilitaron la vinculación o revinculación de HG y MT a la atención y al tratamiento. Esta relación se caracterizó por:

*Trato horizontal*

Los navegadores, mediante una actitud empática y técnicas de escucha activa, fomentaron una relación horizontal que facilitó la colaboración con los usuarios. Esto llevó a intercambiar experiencias acompañando a los usuarios, promoviendo un rol activo para su vinculación o re-vinculación al tratamiento, y concientizándoles sobre la importancia de la adherencia al tratamiento. Miguel, por ejemplo, remarcó el trato personalizado y afectuoso que había recibido del navegador:

.*.. la confianza y el amor a cada uno de los pacientes…. Yo voy a otro consultorio y ni la enfermera te saluda, ni nada. No es igual que [nombre del navegador], ´bienvenido ven pa acá, aquí te lo buscamos tranquilo amigo´, y es esa empatía pues* (Miguel, 28 años, HG, diagnóstico antiguo).

*Soporte emocional y social*

Como parte de la relación establecida con los usuarios, la contención emocional y social brindada por los navegadores durante las sesiones de ARTAS y durante el acompañamiento facilitó el vínculo con el usuario. Este vínculo permitió que los usuarios pudieran expresar sus sentimientos, ayudó a clarificar sus vivencias emocionales y comprender que sus reacciones eran válidas. Este proceso también ayudó a desarrollar autoeficacia en los usuarios para poder afrontar y aceptar su diagnóstico.

Los navegadores abordaron diversas actitudes negativas hacia el VIH en los usuarios derivadas del estigma internalizado, que influían en cómo se autopercibían y creían ser percibidos por su entorno. El estigma internalizado causaba en los usuarios temor o vergüenza de revelar su diagnóstico, y problemas en la adherencia al tratamiento en situaciones de convivencia con personas que desconocían su estado serológico. Esta necesidad de ocultar su diagnóstico convirtió a los navegadores en la única red afectiva y de contención para los usuarios. Así, éstos últimos mostraron gran satisfacción ante el apoyo de los navegadores para seguir adelante y dijeron confiar en ellos para contarles sus preocupaciones o vivencias. La mayoría estableció fuertes vínculos emocionales con los navegadores, refiriéndose a ellos como amigos, familiares o como «alguien enviado del cielo».

*La considero como una amiga, como una mamá. [navegadora] me apoyó en todo. Yo le digo "gracias a ti estoy bien"* (Ricardo, 22 años, HG, diagnóstico reciente).

*Importancia del rol de pares de los navegadores*

Los usuarios manifestaron sentir mayor comodidad por la condición de pares de los navegadores por tener VIH, o en el caso de los navegadores hombres, por compartir su identidad sexual. Esto permitió que pudieran percibir al navegador como un modelo a seguir y aprender, a través de sus experiencias, estrategias para reemplazar el «se viene el mundo abajo» con «yo también puedo estar bien». Las experiencias de los navegadores, viviendo con VIH por mucho tiempo, y quizás también su edad, permitieron a los usuarios disminuir sentimientos de soledad, culpa y fatalismo frente al VIH. Pedro, citado en el título, refiriéndose a compartir su identidad sexual con el navegador, pero también implícitamente al vivir con VIH, expresó:

*Por ser como nosotros, nos entiende. Es nuestro papá gay, nos trata por igual, nos guía. Si conozco a alguien que tiene el mismo diagnóstico le diría que venga, que ahí tengo a [navegador]… él te va ayudar, va a ser tu amigo* (Pedro, 28 años, HG, diagnóstico antiguo).

*Obstáculos en el trabajo de los navegadores*

Pese a la efectividad de su trabajo y la buena relación establecida con los usuarios, en algunos casos los navegadores expresaron frustración ante ciertas barreras evidenciadas en los usuarios como el consumo de drogas (pasta básica de cocaína) o los valores de algunos de ellos o sus familias que consideraban al VIH como «castigo de Dios». Estos comportamientos y creencias despertaban actitudes, sentimientos de culpabilidad y otras emociones negativas que impedían la normalización del diagnóstico, la aceptación de su sexualidad o la vinculación a la atención. Un navegador calificó el rechazo familiar como obstáculo:

*La familia no sabía que era gay y que se infectó, él no iba a hacer su tratamiento para que no se enteren. Tuve que hablar con la mamá desde mi experiencia. Hasta ahora ella dice ‘yo no lo voy a perdonar’, pero siempre que hay visita [médica] lo acompaña. Fue difícil porque a veces las religiones pesan mucho* (Navegador 2).

### Articulación del rol del navegador con el personal de salud

Los navegadores, a través de su trabajo, desarrollaron o fortalecieron su articulación con el resto del personal de salud. Esto les permitió acortar trámites para los usuarios, facilitar la reserva de citas médicas, mejorar el seguimiento y ayudar a fortalecer la relación médico-paciente. Cabe destacar que, aunque al comienzo de la intervención PO+ organizó capacitaciones de sensibilización al personal de salud relacionadas al trato y necesidades de HG y MT, ni PO+ o los establecimientos de salud implementaron cambios para facilitar el flujo de pacientes, trámites o reserva de citas. Solo los navegadores cumplieron ese rol.

Los navegadores manifestaron que el personal de salud mostró satisfacción y apertura hacia su trabajo, confiando en su función y solicitándoles orientar a usuarios sobre los servicios de salud:

*Los médicos me dicen ‘necesitamos que hables con una persona nueva, ayúdalo a entender el sistema’. Converso con él, veo su dificultad. Empezamos con ARTAS, que vea su potencialidad* (Navegadora 2).

Los navegadores ejercieron el rol de nexo entre proveedores de salud y usuarios. Por ejemplo, ellos comunicaban a los proveedores de salud inquietudes o problemas que frecuentemente los usuarios evitaban abordar por timidez o temor a ser juzgados. Una navegadora se refirió a cómo les motivaba a comunicarse mejor con su proveedor:

*Les motivo: ‘Dile la verdad, la doctora no se va a molestar. Los médicos entienden que son cosas íntimas.’ Les sensibilizo, porque piensan ‘qué vergüenza, me va a mirar [partes genito-anales]’* (Navegadora 1).

## DISCUSIÓN

Los resultados sugieren que la navegación implementada por navegadores pares puede ser una estrategia útil para mejorar la vinculación en el CPC de HSH y MT en Perú. Siendo que la navegación de pares representa una compleja intervención a nivel social cuyos elementos o procesos no han sido examinados adecuadamente ^13^, la contribución del presente estudio a la literatura es analizar los elementos relacionados con la implementación del programa de navegación de PO+ en Lima Sur que facilitaron o influenciaron la vinculación a la atención médica.

Los servicios provistos y la educación sobre el VIH, la relación de apoyo de los navegadores con los usuarios y la articulación de su rol con el resto del personal de salud, ejes y temas principales de los resultados, ayudaron a HG y MT a normalizar la condición del VIH y facilitaron su vinculación y revinculación a los servicios de salud del VIH. Los servicios de navegación y las funciones de los navegadores se caracterizaron por un apoyo emocional y acompañamiento personalizado para que los usuarios pudieran identificar o desarrollar fortalezas y superar barreras para manejarse dentro del sistema de salud. Los navegadores desarrollaron relaciones de confianza que promovieron empoderamiento, autoeficacia y cumplieron un rol protector que ayudó a potenciar la resiliencia de los usuarios y se convirtieron en modelos a seguir para el cuidado de la salud y la adherencia al tratamiento viviendo con VIH. Asimismo, los resultados refuerzan la importancia del rol y la influencia de pares al compartir características o experiencias similares conducentes a una mayor empatía y promoción de comportamientos saludables ^29^ donde, en este caso HG y MT, asumen un papel activo en su propio cuidado al vivir con VIH. El trabajo de los navegadores contribuyó a llenar una brecha en los frecuentemente colapsados y poco amigables servicios de atención del VIH, para que los usuarios contaran con una persona con tiempo y esfuerzo exclusivo que los comprendiese para guiarlos en el proceso de vinculación a la atención. Además, los resultados sugieren la factibilidad de incorporar navegadores pares a los equipos de salud con funciones adicionales a las de promotores o consejeros de pares, facilitando el funcionamiento de los servicios y el trabajo del personal médico y contribuyendo a una mayor horizontalidad y participación comunitaria en los servicios para el VIH ^30^.

Los navegadores ayudaron a los usuarios a manejarse en el sistema de salud, pero aún persisten las barreras sistémicas ya mencionadas en la introducción que dificultan la vinculación a la atención. No obstante, promoviendo un rol activo para el cuidado de la salud y desarrollando una toma de conciencia de la realidad de los servicios, este programa de navegación puede contribuir al empoderamiento de PVV y a su vinculación al cuidado médico. Un modelo de empoderamiento implica la construcción de un proceso de involucramiento y compromiso del usuario con su salud, contando con información adecuada y recursos personales para evaluar dicha información, en colaboración con los proveedores de la salud, y con suficiente tolerancia de la incertidumbre en lo que se refiere a la atención médica ^31^.

Este estudio presenta algunas limitaciones. Consistente con métodos cualitativos, es decir, los resultados hallados no son generalizables a todo usuario con VIH, pero son similares a lo encontrado, por ejemplo, en otros estudios sobre programas de navegación con HG en Guatemala y con poblaciones de origen latino en EE. UU que incluían HG y MT ^32^^,^^33^. Los usuarios que se entrevistaron para el presente estudio estaban vinculados a la atención médica, por lo que no tenemos evidencia de las percepciones de HSH y MT que no se vincularon o abandonaron el cuidado. La muestra empleada de usuarios fue intencional, quienes fueron identificados por los navegadores, pero los criterios de inclusión fueron corroborados por las entrevistadoras con los usuarios antes de realizar las entrevistas. Aunque una dificultad del análisis temático podría ser el identificar y priorizar temas y subtemas relevantes, evitamos esa barrera a través de un análisis riguroso, la interrelación temática, y la consistencia y cohesión en la interpretación en equipo de la información. El vínculo navegador-usuario podría ser afectado por distintas razones, aunque eso debería ser evitado con base en la capacitación recibida por los navegadores, considerando que los servicios de navegación están diseñados con una duración determinada y para estar centrados en los usuarios y el desarrollo de sus fortalezas y autoeficacia, resultando en su eventual independencia de los navegadores. Finalmente, no contamos con las percepciones del equipo de salud y proveedores médicos sobre el programa de navegación que podrían ser exploradas en futuros estudios.

Los servicios de navegación pueden tener un valor potencial tanto programático como de costo. Futuras investigaciones podrían examinar específicamente la factibilidad, aceptabilidad y sostenibilidad de los servicios de navegación en Perú, así como los potenciales ahorros de costos resultantes de dicho programa. Los próximos pasos potenciales y desafiantes consistirían en procurar recursos para integrar servicios de navegación para PVV en establecimientos de salud en Perú. Aunque pocos estudios de servicios de navegación realizados en EE. UU. han incluido análisis de costo, sus resultados indican que dichos servicios fueron efectivos a nivel de costos o significaron un ahorro de costos. Su inclusión en servicios de salud en Perú ameritaría también analizar su costo potencial y su efectividad comparado con los servicios estándar actuales ^34^^,^^35^. Asimismo, este programa de navegación se podría aplicar en otros países latinoamericanos con una epidemia del VIH también concentrada en HSH y MT.
